# Influence of the MCT1 rs1049434 on Indirect Muscle Disorders/Injuries in Elite Football Players

**DOI:** 10.1186/s40798-015-0033-9

**Published:** 2015-10-11

**Authors:** Myosotis Massidda, Nir Eynon, Valeria Bachis, Laura Corrias, Claudia Culigioni, Francesco Piras, Paolo Cugia, Marco Scorcu, Carla M. Calò

**Affiliations:** 1 Department of Life and Environmental Sciences, University of Cagliari, Cagliari, Italy; 2 Institute of Sport, Exercise and Active Living (ISEAL), Victoria University, Victoria, Australia; 3 FIMSI CR Sardegna and Cagliari Calcio Spa, Cagliari, Italy

**Keywords:** Elite athletes, Football, Genetic association, Lactate, Muscle injuries, Monocarboxylate transporter-1

## Abstract

**Background:**

The aim of this study was to investigate the association between MCT1 rs1049434 polymorphism and indirect muscle injuries in elite football players. One hundred and seventy-three male elite Italian football players (age = 19.2 ± 5.3 years) were recruited from a first-league football club participating at the Official National Italian Football Championship (Serie A, Primavera, Allievi, Giovanissimi). The cohort was genotyped for the MCT1 rs1049434 polymorphism, and muscle injuries data were collected during the period of 2009–2014 (five football seasons).

**Methods:**

Genomic DNA was extracted using a buccal swab, and genotyping was performed using PCR method. Structural-mechanical injuries and functional muscle disorder were included in the acute indirect muscle injury group.

**Results:**

Participants with the MCT1 AA (AA = 1.57 ± 3.07, *n* = 69) genotype exhibit significantly higher injury incidents compared to participants with the TT genotype (TT = 0.09 ± 0.25, *n* = 22, *P* = 0.04).

**Conclusions:**

The MCT1 rs1049434 polymorphism is associated with the incidence of muscle injuries in elite football players. We anticipate that the knowledge of athletes’ genetic predisposition to sports-related injuries might aid in individualizing training programs.

## Key Points

- MCT1 rs1049434 TT genotype could influence the incidence of muscle injuries in elite football players.
- This study emphasizes a possible future direction in considering genes that encode for monocarboxylate transporters within musculoskeletal soft tissues when examining the individual predisposition of football players to develop muscle injuries.
- Additional research and replication studies are needed to support our data and to fully understand the relationship between predisposition to muscle injuries in football and the MCT1 rs1049434.

## Background

Lactate is the end product of anaerobic glycolysis and is highly accumulated during high-intensity exercise. Lactate transport across the plasma membrane is mainly mediated by proton-linked monocarboxylate transporters (MCT1 and MCT4) [1–3] that play a relevant role in the intracellular pH homeostasis [4]. In fact, the rapid transport of lactic acid across the plasma membrane is fundamentally important for the metabolism of almost all cells, including skeletal muscle.

In 1992, Garcia et al. [5] cloned the first monocarboxylate transporter, MCT1, from Chinese hamster ovary cells and showed that it is expressed, among other tissues, in erythrocytes, skeletal muscle, and heart. Intercellular MCT1 is particularly important in skeletal muscle, where glycolytic fast twitch muscle fibers produce lactate, which is transported out of the cell through the monocarboxylate transporter (MCT). Lactate is then taken up and oxidized by the oxidative slow twitch muscle fibers, which express MCT1 [6]. Kinetic analysis of MCT1 has suggested that it translocates a proton (H^+^) and a monocarboxylate across the plasma membrane by an ordered mechanism in which H^+^ binding precedes monocarboxylate binding and, after translocation across the plasma membrane, the substrates are sequentially released in reverse order [4, 7]. However, it is important to note that the direction of flux is decided by the combined electrochemical gradients of lactate and proton [3].

SLC16A1 is the gene for human MCT1 and it is located on chromosome 1, in position 1p13.2-p12. Defects in SLC16A1 are the cause of symptomatic deficiency in lactate transport, also known as erythrocyte lactate transporter defect. Symptoms and signs of muscle injury on exercise and heat exposure and subnormal erythrocyte lactate transport have been related with the presence of two common single nucleotide polymorphisms (SNPs) in the SLC16A1 gene [8]. In particular, the A > T SNP located in protein coding region (coding base 1470), converting #490 Glutamic amino acid to Aspartic amino acid was associated with a reduction of 35–40 % in the erythrocytes lactate transport rate in two patients carrying the T allele [8].

A recent study showed a higher lactate blood accumulation slope in capillary (more arterialized) blood in T allele carriers than in non-carriers during high intensity (80 %, 15 repetition maximum (15RM)) circuit weight training) [9]. The same research group has recently found a higher venous blood lactate levels in AA genotype carriers compared to carriers of the AT and TT genotypes [10].

Lactate transporter defects in skeletal muscle might provide an explanation for some cases of easy fatigue and muscle cramping upon exercise due to delayed removal of the protons accumulated during (partially) anaerobic work [8]. Muscle fatigue has been shown to predispose to injury [11].

Muscle injury is one of the major disorders that football players are facing. It has been reported that muscle injuries account for 20 to 37 % of all time loss injuries at men’s professional level and 18 to 23 % at men’s amateur level [12, 13]. Muscle injuries are generally classified into two groups; indirect muscle injuries (can also refer as “non-contact” and are caused by internal forces) and direct muscle injuries (caused by external forces). Contusion and laceration are the most common direct muscle injuries. The severity of the direct injury depends on the contact force, the contraction state of the affected muscle at the moment of injury and other factors. However, the functional muscle disorder and the structural muscle injuries are the two group inside which can be classified the indirect muscle injuries (Reviewed by Mueller-Wohlfahrt et al. [20]). Recent studies have identified some genetic markers that could influence the predisposition to injuries in football [14–19]. However, to date, no study has looked at the MCT1rs1049434 variant.

Given the potential role of MCT1 in muscle soreness and injuries, the aim of the present study was to analyze the effect of the MCT1rs1049434 polymorphism on indirect muscle injury rates in top-level football players, during five competitive seasons. We hypothesized that the presence of the T allele (previously associated with greater arterial blood lactate levels) could be protective in developing pathogenesis of indirect muscle injuries due to the decreased transport mediated by MCT1 proteins from arterial blood into the muscle fibers for its oxidation.

## Methods

### Participants

One hundred seventy-three male elite football players (age 19.4 ± 5.2 years; height 177.1 ± 7.4 cm; weight 69.3 ± 8.8 kg) participated in the study. All participants have played at the Official National Football Championship (Serie A, Primavera, Allievi and Giovanissimi) during five seasons (2009–2014). Participants were all Italian (Caucasian) for ≥3 generations. The participants trained for ~8 weeks (25.0 ± 10.3 h per week) in the pre-season period and ~38 weeks during the competitive season (10.0 ± 7.4 h per week). Players who join/left the cohort were included/excluded from the date when they join/left the team. All participants provided informed written consent, and the study protocol was approved by the Ethics Committee of the club and it was in accordance with Declaration of Helsinki for Human Research of 1974 (last modified in 2000).

### Study Design

The study design followed the consensus on definitions and data collection procedures in studies of football injuries outlined in the consensus document [21] and by UEFA [22].

### Injuries Data Collection

Data was collected from 25 players (14.4 %) during the 2009–2010 season, 15 players (8.6 %) during the 2009–2011 season, 14 players (8.0 %) during the 2009–2013 season, 67 players (38.7 %) during the 2012–2013 season, and 53 players (30.6 %) during 2013–2014 season. The follow-up time for injuries was between 1 and 5 years. Injury data of players who left the team during the season, for example, due to a moving to another team, were included only for the period they were part of the roster. Players with an existing injury were not excluded from the study; however, their existing injuries were recorded and were not included in the study.

An indirect muscle injury was defined as any physical complaint occurring during practice that prevented a player from participating in training or match play for at least 1 day after the day of the onset [21]. Injuries were categorized under four degrees of severity based on the number of days’ absence: minimal (code 1; 1–3 days), mild (code 2; 4–7 days), moderate (code 3; 8–28 days), and severe (code 4; >28 days) [21]. The registration of a muscle injury was based on a clinical examination by the team medical staff. Time loss due to injury was recorded on a weekly basis by the team’s medical staff (physicians and coaches) using a standardized injury report form during the preseason and during the regular season.

Ultrasound and magnetic resonance imaging scans were used to morphologically classify the injuries. Muscle injuries included in the study were classified according to a scheme devised by Muller-Wohlfarth [20] derived from a recent consensus statement on sports injuries in relation to indirect mechanisms of injury (Table 1).

**Table 1:** Classification of indirect muscle disorders and injuries

A) Functional muscle disorder	Type 1: Overexertion-related muscle disorder	Type 1a: Fatigue-induced muscle disorder
		Type 1b: Delayed-onset muscle soreness (DOMS)
	Type 2: Neuromuscular muscle disorder	Type 2a: Spine-related neuromuscular muscle disorder
		Type 2a: Muscle-related neuromuscular muscle disorder
B) Structural muscle injury	Type 3: Partial muscle tear	Type 3a: Minor partial muscle tear
		Type 3b: Moderate partial muscle tear
	Type 4: (Sub)total tear	Type 4a: Subtotal or complete muscle tear
		Type 4b: Tendinous avulsion

Participants with direct muscle injury (contusion and laceration) were excluded from the study.

Injury incidence was calculated per 1000 h of training exposure ((∑ number of muscle injuries/∑ h training exposure)×1000). Training exposure was defined as any team-based or individual physical activity, conducted under the control or guidance of the team’s coaching and fitness staff that was aimed at maintaining or improving players’ football skills or physical condition. Matches between teams were considered to be training exposure. Any match activity that was part of a player’s rehabilitation from injury was not recorded as a match exposure. The follow-up time was accounted in the injury incident calculation.

### DNA Analysis

Genomic DNA was extracted from buccal swab using a QIAamp DNA Minikit (QIAGEN, Hilden, Germany). Concentration of extracted DNA was determined by fluorometric method (through Qubit by Invitrogen). On average, the DNA concentration registered was 20 μg/ml, which is sufficient for performing PCR.

Following performing the PCR, an electrophoresis on agarose gel was carried out as quality control to verify the purity of the PCR products. Genomic DNA from the subjects was analyzed by polymerase chain reaction (PCR) following the protocol previously published [9]. The primers used for amplification were as follows [8]: sense primer 5′-ACACATACTGGGCATGTGGC-3′ (1455–1474); antisense primer 5′-AAA TCCCATCAA TGA ACAACTGGTATGATTTCCAC-3′ (1807–1841). Using the primers and the missense polymorphism (rs1049434) described by Cupeiro et al. [9], we searched the sequence on gene bank. Through the Neb Cutter tool (http://tools.neb.com/NEBcutter2/index.php), we individuated the restriction enzyme BccI that allows to discriminate the presence or absence of missense polymorphism A1470T. This enzyme recognizes the sequence 3′-GGTAG-5′ and produces three fragments in the mutate sequence (TT: 14,171, and 202 bp), whilst only two fragments (AA: 14 and 373 bp) are generated in the wild sequence. Heterozigotes AT were individuated by four fragments: 14, 171, 202, and 373 bp.

### Statistical Analysis

Levene’s tests were performed to verify the homogeneity of variances. ANOVA was used to examine the differences between genotype (or allele) groups and continuous data. Where appropriate, a Fisher LSD post hoc analysis was used to determine which of the three genotypes were significantly different from each other.

Hardy-Weinberg equilibrium was calculated using Genepop version 4.0.10 (http://genepop.curtin.edu.au). The *P* value for significance was set at *P* < 0.05.

## Results

The estimated total incidence was 1.25 ± 3.0 muscles injuries per 1000 h of exposure. A total of 107 injuries recorded during the five seasons accounted for a loss of 2391 days, that is, absence from training in the pre-season and regular competitive season. The mean lay-off time for indirect muscle injuries amounted to 13.6 days.

Injuries were most likely to be in the lower extremities (thigh 82.2 %; leg 12.1 %). Muscle injuries in the lower extremities involved the biceps femoris (26.4 %), the rectus femoris (22.9 %), the gastrocnemius (11.4 %), the adductor magnus (11.4 %), the adductor longus (8.0 %), the ileopsoas (5.7 %), the semitendinosus (3.4 %), the semimembranosus (2.2 %), the adductor brevis (2.2 %), the popliteus (1.1 %), the sartorius (1.1 %), the soleus (1.1 %), the vastusmedialis (1.1 %), and the vastusintermedius (1.1 %). Table 2 reports the MCT1 genotype distribution based on the severity of muscle injuries resulting in absence from training sessions or matches.

**Table 2:** MCT1 genotype distribution based on the severity of muscle injuries resulting in absence from training sessions or matches

Degree of severity	Number of injuries	% of total injuries	MCT1 genotype distribution		
			AA (%)	AT (%)	TT (%)
Minimal (1–3 days)	5	4.7	3 (60.0)	2 (40.0)	0 (0)
Mild (4–7 days)	19	17.8	10 (55.5)	8 (44.4)	0 (0)
Moderate (8–28 days)	56	52.3	15 (41.6)	19 (52.7)	2 (5.5)
Severe (>28 days)	27	25.2	15 (60.0)	8 (32.0)	2 (8.0)

Table 3 reports the number and percentage of muscle injuries classified according to the Munich Consensus Statement.

**Table 3:** Muscle injury classification according to the Munich consensus statement

Type of injury	Number of injuries	% of total injuries	MCT1 genotype distribution number of athletes (%)		
			AA (%)	AT (%)	TT (%)
Overexertion-related muscle disorder	13	12.2	5 (55.5)	3 (33.3)	1 (11.1)
Neuromuscular muscle disorder	62	57.9	23 (52.2)	19 (43.1)	2 (4.5)
Minor partial muscle tear	21	19.6	9 (64.2)	5 (55.5)	0 (0.0)
Moderate partial muscle tear	10	9.4	4 (44.4)	5 (55.5)	0 (0.0)
Sub(total) muscle tear	1	0.9	0 (0)	1 (100.0)	0 (0.0)

The MCT1rs1049434 genotype distribution (39.8 % AA, *n* = 69; 47.3 % AT, *n* = 82; 12.7 % TT, *n* = 22) was in Hardy-Weinberg equilibrium (*P =* 0.486) confirming no genotyping errors, population subdivision, viability selection, or systematic mating. Age (*P* = 0.322), height (*P* = 0.366), and weight (*P* = 0.353) were not significantly differentiated between the three genotype groups as well as the number of seasons played by the football players (*P* = 0.060) (Table 4).

**Table 4:** General characteristics of the football players when divided into the three MCT1 rs1049434 genotype (AA, AT, TT) groups

	AA (*n* = 69)	AT (*n* = 82)	TT (*n* = 22)	*P* value
Age (years)	20.3 ± 5.5	19.0 ± 5.5	18.9 ± 4.8	0.322
Height (cm)	178.7 ± 7.4	176.5 ± 7.9	176.2 ± 7.1	0.366
Weight (kg)	71.6 ± 7.7	68.5 ± 9.5	68.0 ± 9.3	0.353
Seasons played (months)	18.3 ± 12.0	17.0 ± 11.0	16.7 ± 9.3	0.060

Age, height, weight, and seasons played are expressed as an average ± standard deviation

Regarding the muscle injury variables, significant differences were found between MCT1 genotypes and incidence of muscle injuries (*P* = .048). In detail, football players with the TT genotype had lower incidence of muscle injuries compared to AA genotype carriers (AA = 1.57 ± 3.07, *n* = 69; TT = 0.09 ± 1.25; *P* = .044). No significant difference was found between genotypes and the severity of injuries (*P* > 0.05). Moreover, there was no correlation between the number of the recovery days and the SNP.

Finally, no significant difference between injuries from muscles that are primarily slow twitch compared to muscles that are primarily fast twitch was observed. In fact, the most injured muscles were the biceps femoris and rectus femoris that are primarily slow twitch and fast twitch, respectively.

## Discussion

The main finding of the present study was that the MCT1 rs1049434 AA genotype is associated with higher incidence of injuries in elite football players. The mean incidence of indirect muscle injuries shown in the present study is in line with the recent published data (1.48; range 1.42 to 1.55) [23] on epidemiological and clinical muscle injuries in male elite football players (UEFA Elite League study of 2287 thigh injuries (2001–2013)).

Lactic acid is mainly dissociated into La^−^ anions and protons (H^+^) at physiological pH status. During exercise and muscle contractions, muscle and blood anions (La^−^) and protons (H^+^) are typically raising up, depending on the exercise intensity [24, 25]. Lactic acid transport across the plasma membrane is fundamental for the metabolism and pH regulation of all cells, removing lactic acid produced by glycolysis and allowing uptake by those cells utilizing it for gluconeogenesis (liver and kidney) or as a respiratory fuel (heart and red muscle). Previous researches have argued that any detrimental effects of lactic acid on muscle and exercise performance are due to H^+^ rather than La^−^ [26], even if other evidences have given alternative explanations of the biochemistry of metabolic acidosis [27]. Muscles predominantly composed of oxidative fibers (e.g., soleus) express considerable amounts of MCT1 and very little MCT4 [28, 29], whereas muscles with a large proportion of fast twitch, glycolytic fibers (e.g., white gastrocnemius) express primarily MCT4 and very little MCT1, which have different kinetic properties [29]. Both MCT1 and MCT4 participate in the cell–cell lactate shuttle, whereas MCT1 facilitates operation of the intracellular lactate shuttle [30].

Cupeiro et al. [9] have shown an association between the MCT1 rs1049434 polymorphism and lactate accumulation; higher capillary lactate accumulation was noted in men carrying the T allele during high-intensity circuit training. More recently, Cupeiro et al. [10] published another study in which they found a higher lactate accumulation in AA males, which would reflect a higher release of this molecule from the active fiber to the blood. Nevertheless, with the data available, the authors cannot conclude if the differences found between the two studies are due to the different blood type analyzed (capillary vs. venous), different training protocols used, or different muscle fiber proportions within each individual [31].

On the bases of the accepted “cell-to-cell lactate shuttle” model, the lactate movement across the sarcolemma during exercise is mediated by monocarboxylate transporters MCT1and MCT4 that are primarily responsible for lactate uptake from the circulation and lactate extrusion out of muscle, respectively [32]. Therefore, if we consider our results within this model, we could hypothesize that the highest incidence of muscle injuries displayed by football players with AA genotype could be due to acidic intracellular environment created by muscle activity due to the elevated lactate transport from arterial blood into the muscle fibers for its oxidation, as noted in the Cupeiro at al. study [10].

The possible link between muscle injuries and lactate level could be sought in the variations in lactate transporter that results in an acidic intracellular environment created by muscle activity with consequent degeneration of muscle and release of myoglobin and creatine kinase [33–35]. This factor might compromise extreme performance in healthy individuals. Considering that muscle fatigue has been shown to predispose to injury [11], lactate transporter variations in skeletal muscle might provide an explanation for some cases of muscle injuries due to the higher intramuscular lactate concentration, and genetic variations can explain some of the predisposition to injuries.

The primary limiting factor in genetic association studies is the need to recruit large groups of cases (elite athletes in this case) to overcome the obvious barrier of large sample size for detecting genetic associations. In regard to that, we stress that our sample is extremely homogeneous in terms of the level of the players (all elite-level football players), geographical origin of the participants (all Italians and Caucasians), sex (all males), and team (all from the same team). Hence, their exposure to training loads and injuries and other confounding variables are well controlled.

A specific limitation in the present study is lack of data on intramuscular lactate concentration that could, potentially, assist clarifying the physiological mechanism behind the genotype:phenotype association. Although our study may pave the way in looking at this specific SNP as potential genetic marker, more muscle-related injury and replication studies in larger cohorts as well as studies with more injury-related phenotypes are required to confirm this association.

## Conclusions

In conclusion, we found that the MCT1 rs1049434 polymorphism is associated with the incidence of muscle injuries in elite football players. Additional research is required to support our data and to fully elucidate the relationship between predisposition to muscle injuries in football and the MCT1 rs1049434 polymorphism.
